# Alzheimer’s Disease and Paraoxonase 1 (*PON1*) Gene Polymorphisms

**DOI:** 10.2174/1874091X01711010047

**Published:** 2017-06-13

**Authors:** Mohsen Saeidi, Raheleh Shakeri, Abdoljalal Marjani, Safoura Khajeniazi

**Affiliations:** 1 Stem Cell Research Center, Gorgan Faculty of Medicine, Golestan University of Medical Sciences, Gorgan, Golestan province, Iran; 2 Student Research Committee, Gorgan Faculty of Medicine, Golestan University of Medical Sciences, Gorgan, Golestan province, Iran; 3 Metabolic Disorders Research Center, Department of Biochemistry and Biophysics, Faculty of Medicine, Golestan University of Medical Sciences, Gorgan, Golestan province, Iran; 4 Department of Medical Technology, Faculty of Advanced Medical Sciences and Technology, Golestan University of Medical Sciences, Gorgan, Golestan province, Iran

**Keywords:** PON1, Gene, Polymorphism, Alzheimer, Disease, Gorgan

## Abstract

**Background:**

Some studies have indicated that human paraoxonase 1 (*PON1*) activity shows a polymorphic distribution. The aim of this study was to determine the distribution of *PON1* polymorphism in patients with Alzheimer’s disease in Gorgan and compare it with a healthy control group.

**Method:**

The study included 100 healthy individuals and 50 patients. Enzyme activity and genetic polymorphism of *PON1* were determined.

**Result:**

There were significant differences in distribution of genotypes and alleles among patients and control group. The most common genotype was CT in patients and control group, while the most frequent alleles were T and C in patients and controls, respectively. There was a statistically significant variation between serum *PON1* activity and –108C> T polymorphism. The highest *PON1* enzyme activities in the patients and controls were found in CC, while lower enzyme activities were seen in CT and TT genotypes in both genders and age groups.

**Conclusion:**

Onset of Alzheimer’s disease may depend on different polymorphisms of the *PON1* enzyme. Late or early-onset of Alzheimer’s disease may also depend on age and gender distribution, especially for arylesterase enzyme. Further studies on polymorphism of the enzyme are necessary for interpretation of possible polymorphic effects of enzyme on *PON1* activity in humans.

## 1 INTRODUCTION

Alzheimer’s disease (AD) is the most common type of dementia [1]. It affects one in eight individuals aged over 60 years [2]. The prevalence of dementia is increasing and is expected to reach 24 million by the year 2040 [3, 4]. The increasing prevalence of AD has been reported in some countries [5]. A study has shown an association between *paraoxonase 1* (*PON1*) activity and the pathogenesis of AD [6]. Human *PON1* exhibits both paraoxonase and arylesterase activities. It hydrolyzes organophosphate compounds such as paraoxon, and aromatic carboxylic acid esters [7-10]. *PON1* is associated with high-density lipoprotein (HDL) [11]. The enzyme reduces accumulation of the lipid peroxides in low-density lipoprotein (LDL) [12]. *PON1* is a main anti-atherosclerotic component of high-density lipoprotein (HDL) [13, 14]. *PON1* also protects against bacterial infection by destroying the bacterial signalling molecules [15]. The *PON1* polymorphism is associated as risk factor for neurological diseases [16-18]. Several studies have shown that *PON1* status and oxidative stress could play important roles in many neurodegenerative diseases [16, 19-25]. More than 160 polymorphisms have been shown in the *PON1*gene [26]. Study of Primo-Parmo *et al.* revealed that *PON1* included three genes: *PON2, PON3* and *PON1* that are located on the long arm of human chromosome 7 (q21.22) [27]. The expression of *PON* gene family members occurs in different types of tissues in the human body [27]. The *PON3* and *PON1* genes, and *PON2* gene are expressed and synthesized in the liver and various tissues (brain, liver, kidney, and testis), respectively [28]. Secreted *PON1* and *PON3* enzymes from liver cells are found in the blood circulation bound to high-density lipoproteins, while *PON1* activity predominates in human serum [29]. The *PON2* enzyme synthesized in many tissues is not released from the cells [21]. The *PON1* is the best investigated and described member of the family [30]. Several polymorphisms of *PON1* gene have been reported. These polymorphisms may be associated with *PON1* expression and enzyme activity [31-38].

Different distribution of *PON1* gene polymorphism makes these polymorphisms important in different ethnic groups. Thus, the aim of this study was to determine the distribution of *PON1* polymorphism in patients with AD and compare it with a control group.

## 2 MATERIALS AND METHOD

### 2.1 Study Subjects and Sample Collection

The study included 100 healthy unrelated individuals (63 men and 37 women) and 50 Alzheimer’s patients with late-onset form of the disease (31 men and 19 women). The mean age of Alzheimer’s patients and control group was 75.01± 69.09 and 74.06± 66.10 years, respectively. The patients were directed to an elderly nursing home in Gorgan, Iran. Patients with type 2 diabetes mellitus, liver disease, renal failure and chronic infectious disease were excluded. The control group was selected from the close relatives of Alzheimer’s patients. Both groups were matched in terms of age and gender. The study was approved by the Ethics Committees of Deputy of Research at Golestan University of Medical Sciences. Alzheimer’s patients were diagnosed by a neurologist using MMSE (Mini Mental State Examination) [32]. Written consent was obtained from close relatives of all subjects. Ten ml blood samples were collected in EDTA-tubes and serum tubes for determination of *PON1* genotypes and *PON1* activity, respectively. Collected samples were stored at −20°C until analysis.

### 2.2 Determination of *PON1*

Determination of serum paraoxonase and arylesterase activities were carried out by Brophy *et al.* method [33] using spectrophotometry technique (Model JENWAY 6105 UV/VIS) at the Metabolic Disorders Research Center, Golestan University of Medical Sciences.

### 2.3 Polymerase Chain Reaction (PCR) and Restriction Fragment Length Polymorphism Analysis (PCR-RFLP)

White blood cells were used for DNA extraction by salting-out method [34]. Extracted DNA was dissolved by sterilized distilled water. The PCR and PCR-RFLP techniques were used to determine the polymorphism of -108C>T using Genetix CG palm-thermocycler (India). Amplification was performed for DNA fragment containing polymorphic site –108C>T. A 25 μl reaction mixture was prepared for the PCR process including buffer (200 mM Tris-HCl, pH = 8.4 and 500 mM KCl, 1.5 mM MgCl_2_) (Fermentas), 0.3 mM deoxyribonucleotide triphosphate (dNTP), 0.4 U/μl Taq polymerase (Fermentas), 0.3 μM of each primer (Bioneer), 20 ng genomic DNA and 11.1 μL sterile distillated water. Digestion of PCR products (32μl) was performed by restriction enzymes, BsrBI (Fermentas) at 37^o^C for 16 hours. The PCR amplification conditions included initial denaturation (35 cycles at 95^o^C for 3 minutes), denaturation (at 94^o^C for 30 seconds), annealing (at 68^o^C for 30 seconds), extension (at 72C for 60 seconds) and final extension (at 72^o^C for 7 minutes). Figs. (**1** and **2**) show the PCR products before and after digestion with the restriction enzyme (BsrBI), respectively. Agarose gel (2%) stained with ethidium bromide (0.5 μg/ml) was used for electrophoresis of DNA fragments (Apelex, France). The bands were detected using a Polaroid Gel Camera. In Fig. (**1**), undigested fragment (240 bp) was detected. In Fig. (**2**), digested fragment (212 bp) was detected for C-108 genotypes CC, CT and TT. Detection of mutations was performed using the following primers:

Forward primer: 5' AGCTAGCTGCCGACCCGGCGGGGAGGAG 3'

Reverse primer: 5' GGCTGCAGCCCTCACCACAACCC 3'.

### 2.4 Statistical Analysis

SPSS software version 16 was used for data analysis (SPSS Inc., Chicago, IL, USA). The Chi-square test was used to compare allele and genotype frequencies. The Kolmogorov-Smirnov test was used to check the normality of the distribution for *PON1* activity. The Kruskal-Wallis test was used to compare *PON1* activity and genotype. Comparisons of *PON1* activity with genotype distribution of Alzheimer’s patients and control group were analyzed by the Mann-Whitney test. P-value of less than 0.05 was considered as statistically significant.

## 3 RESULTS

This study revealed the *PON1* activity and the genotype and allele frequencies for 108C>T polymorphism of this gene in Alzheimer’s patients and healthy controls. The frequencies of *PON1* genotypes and alleles are shown in Table (**1**). There were significant differences in distribution of –108C>T genotypes and alleles among Alzheimer’s patients and the control group (*P* = 0.03). The most common genotype was CT in the patients (54%) and controls (60%), while polymorphism frequencies of both CC and TT genotypes were lower. The most frequent alleles were T (59%) and C (55%) in Alzheimer’s patients and controls, respectively. Table (**2**) shows the association between *PON1* enzyme activity and promoter region polymorphism in both groups. Table (**2**) indicates that there is statistically significant variation between serum *PON1* activity and –108C>T polymorphism. Tables (**3** and **4**) show the *PON1* enzyme activity in association with promoter region polymorphism in Alzheimer’s patients, in terms of gender and age. The highest PON1 enzyme activity was found for CC in the patients and controls, while lower enzyme activities were observed for CT and TT genotypes in both genders and age groups.

## 4 DISCUSSION

Some studies have indicated that human *PON1* activity showed a polymorphic distribution. This polymorphism could be identified subjects with different paraoxonase 1 activity [26]. Gene frequencies may vary among different ethnic groups [39]. It is shown that *PON1* activity may change up to 40-fold in some population [26, 39, 40]. Thus, it is important to determine the association between genetic polymorphism and status of the *PON1* gene in Alzheimer’s patients. Our study showed the activity, genotype and allele frequencies for 108C>T polymorphism in healthy controls and Alzheimer’s patients. The present study confirms that *PON1* activity is significantly lower in Alzheimer’s patients compared to controls. Low arylesterase activity may be a predictive risk factor for this disease. Pola *et al*. [41] and also Shi *et al*. [42] in a different study on Chinese population did not find any difference in enzyme activity of Alzheimer’s patients and controls. Helbecque *et al*. [43] and Cellini *et al*. [44] emphasized the importance of promoter -108C>T polymorphism, which may be a risk factor for AD. It was shown that there is an association between the *PON1* gene promoter polymorphism and *PON1* activity [45]. Although it is not clear how the mechanism of *PON1* affects risk of AD, studies have revealed an association between low *PON1* activity, elevated oxidative stress, and increased risk of cardiovascular disease, stroke, type 2 diabetes and dementia [46-49]. Many studies have revealed the association between *PON1* polymorphism and AD [44, 50-56], while some studies have found no association between *PON1* polymorphism and AD in African Americans or Caucasians [50]. The results of the present study showed a significant association between *PON1* polymorphism and risk of AD, which is in agreement with other studies [44, 50-56]. Inconsistent with our findings, research on patients with AD has shown that there is no association between *PON1* polymorphism and the development of the disease [57, 58]. *PON1* may have a protective role in patients with AD [59]. Studies on other neurodegenerative diseases (Multiple sclerosis and Amyotrophic lateral sclerosis) have revealed no association between *PON1* polymorphism or activity and these diseases. Findings of some studies have indicated that *PON1* may play an important role in the pathogenesis of neurological disorders [59, 60]. In the present study, *PON1* polymorphism significantly affected *PON1* activity. *PON1* activity was the highest in CC and the lowest in TT genotype of promoter -108C>T polymorphism. Our results were in accordance with other findings [35, 61]. In this study, the -108 polymorphism revealed a significant difference in Alzheimer’s patients compared to controls (T allele was more frequent). Some studies have shown that -108 region is the only position that does not differ in allele frequency, between white and Japanese populations. This indicates that this polymorphism may show no specific differences in allele frequencies across different ethnic groups [35], which is not in agreement with our study. Some other findings suggested that the -*108* polymorphism shows the greatest effect on arylesterase activity. This polymorphism may be associated with activity variance that is independent from the -108C site [35]. There is a relationship between the -108C allele and the PON1 genotype and disease. The -108 regulatory-region polymorphism has an important effect on *PON1* expression in humans [35]. Our study showed a significant association between *PON1* polymorphism and *PON1* activity among different gender and age groups. The highest enzyme activity was observed in the CC genotype. Alzheimer’s patients older than 70 years have lower enzymatic activity (arylesterase and paraoxonase activities) than patients under 70 years old (except for arylesterase enzyme activity for age above 70 years old). This means that the disease may not begin late in life. Our study also showed that the *PON1* activity in different genotypes of enzyme was lower in women than in men with AD. This means that women may be more susceptible to this disease compare to men. *PON1* promoter polymorphism may affect *PON1* expression. The association of the CC genotype with high *PON1* activity has been reported to be stronger than the TT genotype [38], which is in agreement with our study. Our findings confirmed that the *PON1* gene polymorphism affect serum *PON1* activity. This may indicate a possible association between *PON1* gene polymorphism with the progression of AD in the study subjects. Some studies suggested that *PON1* does not cross the blood-brain barrier. Paraoxonase may express in brain or enter pathways that can disturb the brain [62]. Limited sample size is one of the limitations of the present study, because of small number of eligible Alzheimer’s patients in the elderly nursing home for this study. Our study subjects had not fasted before sample collection and the patients were not under any therapeutic regimen.

## CONCLUSION

Onset of AD may depend on different polymorphism of enzymes, age and gender distribution. Further studies on polymorphism of enzymes are necessary for interpretation of possible polymorphic effects of enzyme on *PON1* activity in humans.

## Figures and Tables

**Fig. (1):**
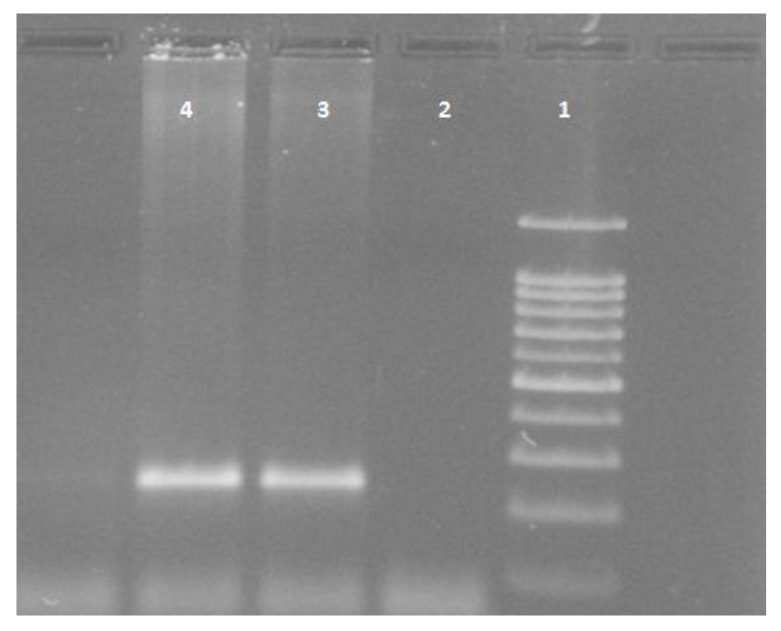
Determination of undigested –108C>T polymorphism (240bp) by PCR-RFLP. DNA ladder (100bp) was loaded into well 1; well 2 was negative control; wells 3 and 4 were DNA fragments with 240bp.

**Fig. (2):**
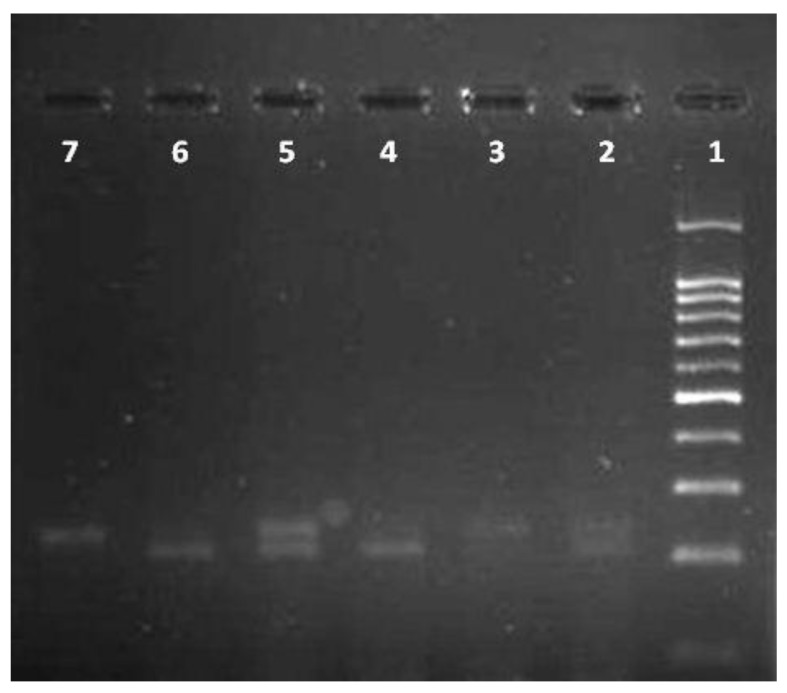
PCR-restriction enzyme (*Bsr*BI digestion) fragmentation patterns on the agarose gel stained with ethidium bromide for determination –108C>T polymorphism. DNA ladder (100bp) was loaded into well 1; wells 2 and 5 were CT; wells 3 and 7 were TT, wells 4 and 6 were CC.

**Table 1:** Genotype and allele frequency of –108C>T polymorphism in Alzheimer’s patients and control group.

Polymorphism	Genotype N=150	Frequency		Allele N=300	Frequency	
		*n*	%		*n*	%
-108C>T (Alzheimer patients)	CC CT TT	7 27 16	14 54 32	C T	41 59	41 59
108C>T (Control)	CC CT TT	25 60 15	25 60 15	C T	110 90	55 45
P value	0.03					

Data are shown as percentage, examined by the χ^2^ test.

**Table 2:** *PON1* enzyme activity in association with promoter region polymorphism in Alzheimer’s patients and control group.

Enzymes Activity (IU/A)	Genotypes (Alzheimer patients)			Genotypes (control)		
	CC CT TT			CC CT TT		
Paraoxonase	45.42*	27.20*	13.9*1	76.44	43.07	37.0
Arylesterase	41.0*	25.81*	18.06*	77.56	44.45	29.66

*P< 0.001 (Data are shown as the median, examined by the Mann-Whitney test).

(IU/L= 1 international unit of enzyme activity is explained as enzyme catalyzes the reaction.

rate of 1 μmol per minute in an assay system).

**Table 3:** *PON1* enzyme activity in association with promoter region.

Genotype	n	Mean Rank	
		Paraoxonase Activity (IU/L)	Arylesterase Activity (IU/L)
**Men(n=31)** CC CT TT P value	22 50 23	72.32 43.59 34.33 < 0.001	74.50 44.55 30.15 < 0.001
**Women (n=19)** CC CT TT P value	10 37 8	37.70 28.66 12.81 0.009	38.35 27.96 12.25 0.004

Data are shown as the median, examined by the Kruskal-Wallis and Mann-Whitney tests.

**Table 4:** *PON1* enzyme activity in association with promoter region.

Genotype	n	Mean Rank	
		Paraoxonase IU/L Activity	Arylesterase U/L Activity
**≤70 (n=31)** CC CT TT P value	28 52 12	66.79 40.09 26.96 < 0.001	67.68 39.92 25.58 < 0.001
**>70 (n=19)** CC CT TT P value	4 35 19	39.00 32.80 21.42 0.01	43.00 32.80 21.42 0.3

Data are shown as the median, examined by the Kruskal-Wallis and Mann-Whitney tests.
